# Effect of Osseodensification on Ridge Expansion and Bone Density for Implant Placement in the D3 and D4 Bone of the Posterior Maxilla: A Comparative Study

**DOI:** 10.7759/cureus.113245

**Published:** 2026-07-23

**Authors:** Ritu Raj Gupta, Jogeswar Barman, Bhawana Bhawana, Shireen L Dkhar, Pranjal Charingia

**Affiliations:** 1 Department of Prosthodontics and Crown &amp; Bridge, Regional Dental College, Guwahati, IND; 2 Department of Prosthodontics and Crown &amp; Bridge, Regional Dental College and Hospital, Guwahati, IND; 3 Prosthodontics, Regional Dental College Guwahati, Guwahati, IND

**Keywords:** bone density, cone-beam computed tomography, d3 bone, d4 bone, densah burs, dental implants, osseodensification, posterior maxilla, ridge expansion

## Abstract

Background

Implant placement in the posterior maxilla is often challenging because of reduced bone density, limited ridge width, and compromised primary stability, particularly in D3 and D4 bone. Conventional osteotomy preparation is subtractive and may further reduce available trabecular bone support. Osseodensification is a bone-preserving osteotomy technique that compacts autogenous bone along the osteotomy walls and may improve peri-implant bone density and ridge dimensions. This study aimed to evaluate the effect of osseodensification on alveolar ridge expansion and peri-implant bone density during implant placement in the D3 and D4 bone of the posterior maxilla.

Methodology

An institution-based, comparative clinical study was conducted among 20 patients requiring implant-supported fixed prosthetic rehabilitation in the posterior maxilla. Patients were categorized into D3 and D4 bone groups based on preoperative cone-beam computed tomography (CBCT) assessment. Implant osteotomy was performed using Densah burs according to the osseodensification protocol. Peri-implant bone density and alveolar ridge width were measured at the crestal, mid-apical, and apical levels using CBCT at preoperative, immediate postoperative, and four-month postoperative intervals. Intergroup and intragroup comparisons were performed using appropriate statistical tests, with significance set at a p-value <0.05.

Results

The mean peri-implant bone density in D3 bone increased from 481.5 ± 99.5 HU preoperatively to 578.8 ± 150.0 HU immediately postoperatively and 689.4 ± 193.7 HU at four months. In D4 bone, the mean peri-implant bone density increased from 332.9 ± 78.1 HU preoperatively to 466.7 ± 80.0 HU immediately postoperatively and 588.7 ± 105.6 HU at four months. The intragroup increase in peri-implant bone density was statistically significant in both groups. The mean alveolar ridge width increased from 8.2 mm to 9.8 mm in D3 bone and from 8.0 mm to 8.8 mm in D4 bone. The ridge width gain was more evident at the mid-apical and apical levels than at the crestal level. No significant correlation was observed between age or gender and the evaluated parameters.

Conclusions

Osseodensification significantly improved peri-implant bone density and produced measurable alveolar ridge expansion in both D3 and D4 bone of the posterior maxilla. The technique appears useful for implant site preparation in low-density posterior maxillary bone. Further controlled clinical studies with larger sample sizes and longer follow-up are required to validate these findings.

## Introduction

Dental implants have become a predictable treatment option for replacing missing teeth because they provide improved stability, function, and patient comfort compared with conventional removable prostheses [1]. Their long-term success depends largely on osseointegration, which is influenced by implant material, implant surface characteristics, implant design, host bone quality, surgical technique, and loading conditions [2]. Among these factors, bone quality and quantity are particularly important because adequate bone volume allows proper implant positioning, while bone density contributes to mechanical engagement and primary stability [3].

The posterior maxilla remains one of the most challenging regions for implant placement. This region commonly presents with low-density trabecular bone, reduced ridge dimensions following tooth loss, and maxillary sinus pneumatization [4]. D3 and D4 bone types are frequently encountered in this area and are associated with reduced mechanical resistance during implant insertion [3]. As a result, achieving adequate primary stability may be difficult, especially when conventional subtractive drilling techniques are used.

Conventional implant osteotomy preparation removes bone to create space for the implant fixture. Although this method is effective in dense bone, it may be less favorable in low-density bone because removal of additional trabecular bone can further reduce the mechanical support surrounding the implant bed [5]. Various alternative techniques, including undersized drilling, osteotome condensation, ridge expansion, and guided bone regeneration, have been used to improve implant placement in compromised ridges. However, these methods may be associated with additional surgical trauma, increased patient discomfort, risk of cortical plate fracture, higher treatment cost, or longer healing time [6].

Osseodensification was introduced as a non-subtractive implant site preparation technique designed to preserve and compact bone during osteotomy preparation [7]. This technique uses specially designed densifying burs in a counterclockwise mode under copious irrigation. The burs compact autogenous bone particles along the osteotomy walls, thereby increasing local trabecular density and improving the mechanical environment for implant placement [7]. Lahens et al. reported that osseodensification improved biomechanical fixation and histologic bone response around endosteal implants in low-density bone [8]. Koutouzis et al. demonstrated that osseodensification-mediated plastic deformation can produce measurable ridge expansion and allow simultaneous implant placement [9]. Salman and Bede also reported ridge expansion with osseodensification in narrow alveolar ridges without dehiscence or fenestration [10].

Although previous studies have evaluated osseodensification in relation to implant stability, bone density, and ridge expansion, limited clinical evidence is available regarding its combined effect on peri-implant bone density and alveolar ridge width specifically in the D3 and D4 bone of the posterior maxilla. Furthermore, many studies have assessed ridge changes at limited points, whereas evaluating crestal, mid-apical, and apical levels may provide a more complete understanding of dimensional changes around the implant site. Therefore, the present study was conducted to evaluate the effect of osseodensification on ridge expansion and peri-implant bone density in the D3 and D4 bone types of the posterior maxilla using cone-beam computed tomography (CBCT). We hypothesized that osseodensification would result in significant increases in alveolar ridge width and peri-implant bone density following implant placement.

## Materials and methods

Study design and setting

An institution-based study was conducted in the Department of Prosthodontics and Crown & Bridge, Regional Dental College, Guwahati, Assam, India, affiliated with Srimanta Sankaradeva University of Health Sciences, Guwahati. The study was approved by the Institutional Ethics Committee of the Regional Dental College, Guwahati (approval number: RDC/29/2011/Pt.-I/1746). The study was conducted from August 2024 to January 2026. The study included patients reporting to the Department of Prosthodontics and Crown & Bridge with partial edentulism in the posterior maxillary region who opted for implant-supported fixed prosthetic rehabilitation.

Before inclusion, all patients were informed about the purpose, nature, and design of the study. The implant placement procedure, radiographic assessments, possible surgical risks, expected benefits, postoperative instructions, and follow-up requirements were explained to each patient. Written informed consent was obtained from all participants before enrollment.

Study sample and grouping

A total of 20 subjects were included in the study and categorized into two groups according to the preoperative CBCT assessment of bone quality. Group 1 included 10 patients with D3 bone at the proposed posterior maxillary implant site, while Group 2 included 10 patients with D4 bone.

Inclusion criteria

Patients aged ≥18 years with partial edentulism requiring placement of at least one dental implant in the posterior maxilla were included in the study. Only patients with an edentulous period of at least three months following extraction were considered. The implant site was required to have D3 or D4 bone quality, a residual alveolar bone height of ≥12 mm from the nearest anatomical landmark to the crest (or 1-2 mm above the crest), and a minimum ridge width of 4 mm throughout the implant site length. Sites free from local infection, cystic lesions, or other pathological abnormalities were selected. All participants were required to provide informed consent and demonstrate willingness to undergo surgery, adhere to postoperative instructions, and attend follow-up visits.

Exclusion criteria

Patients with American Society of Anesthesiologists (ASA) physical status III or higher, pregnant or lactating women, and individuals with parafunctional habits such as bruxism or clenching were excluded. Chronic smokers or tobacco users, patients with traumatic occlusion, insufficient inter-arch space for prosthetic rehabilitation, active oral infection, untreated periodontal disease, or poor oral hygiene were also excluded. Additionally, any local or systemic condition that could compromise healing or implant success was considered an exclusion criterion. Patients unwilling to provide consent or unable to comply with the follow-up protocol were not included.

Preoperative assessment

A detailed case history was recorded for each patient, including demographic details, medical history, dental history, history of extraction, oral habits, and general clinical findings. A thorough intraoral examination was performed to assess the edentulous area, adjacent teeth, mucosal condition, oral hygiene status, and available prosthetic space. Preoperative CBCT was performed for diagnosis and treatment planning.

**Figure 1:**
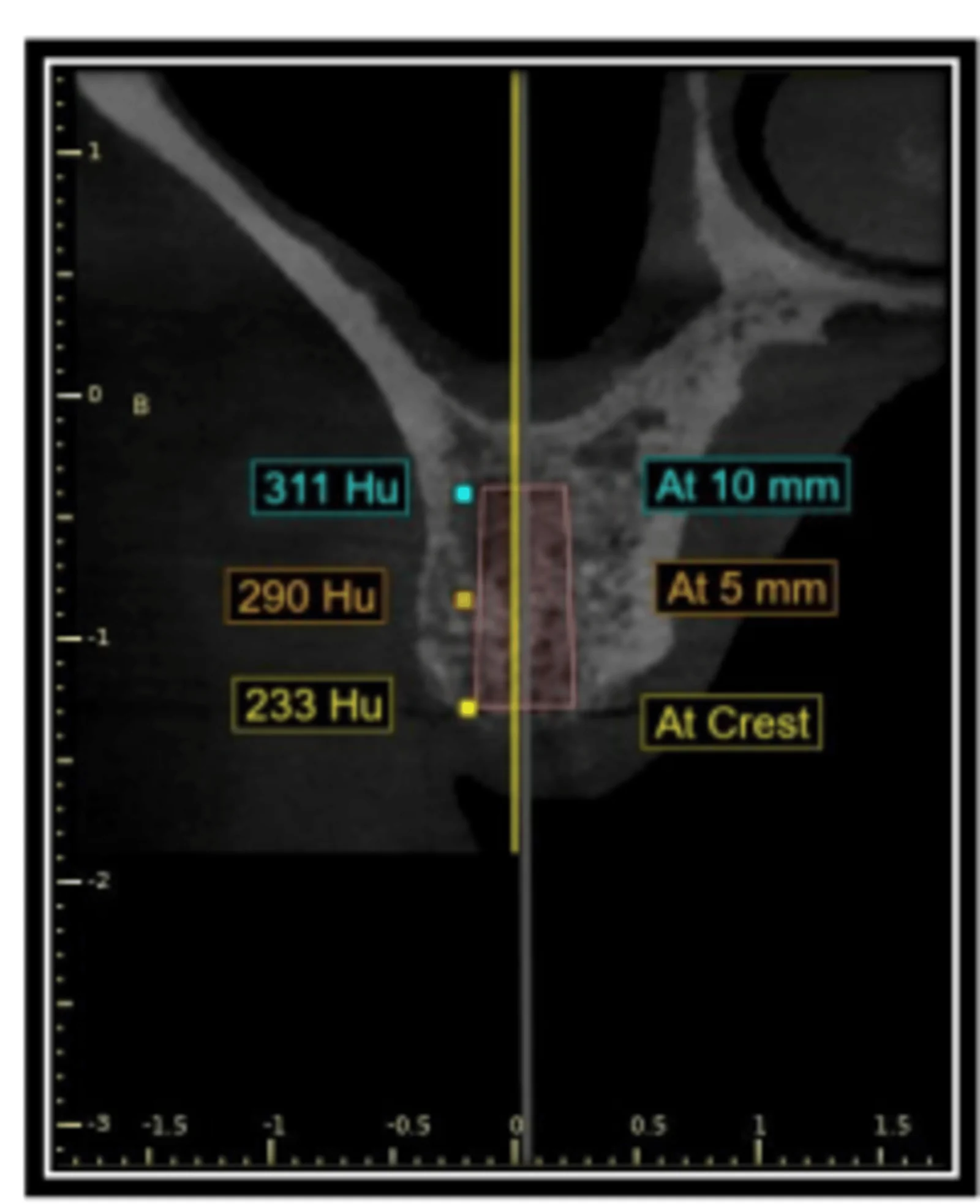
Representative cone-beam computed tomography image showing assessment of peri-implant bone density and alveolar ridge width in the posterior maxilla at L1, L2, and L3 levels. L1 = at the crestal level; L2 =  5 mm above L1; L3 = 5 mm above L2

Bone quality was assessed using gray-scale values obtained from CBCT and expressed in Hounsfield units (HU). Alveolar ridge width and alveolar bone height were measured at the proposed implant site. Radiographic evaluation was performed using the Carestream CS 9000 3D imaging system, with a 4 × 4 cm field of view, 120 kVp, 6.3 mA, 0.18 × 0.18 × 0.18 mm voxel size, and an effective radiation dose of approximately 150 μSv. Standardized imaging parameters were maintained for all patients, and measurements were performed using Blue Sky Plan software (Figure 1).

All radiographic measurements were performed by a single examiner using a standardized measurement protocol to minimize inter-examiner variability.

Surgical protocol

All implant placement procedures were performed under strict aseptic conditions. The operative site was prepared using routine sterilization and draping procedures. Local anesthesia was administered using 2% lignocaine with adrenaline 1:80,000. A mid-crestal incision with a vertical releasing incision was placed at the proposed implant site, followed by elevation of a full-thickness mucoperiosteal flap using Molt’s No. 9 periosteal elevator. Dentis System implants (4.0 × 10 mm), featuring a tapered root-form threaded design with an SLA surface, were used in all patients. All implants were inserted with a final insertion torque of 35 Ncm, following which cover screws were placed.

**Figure 2:**
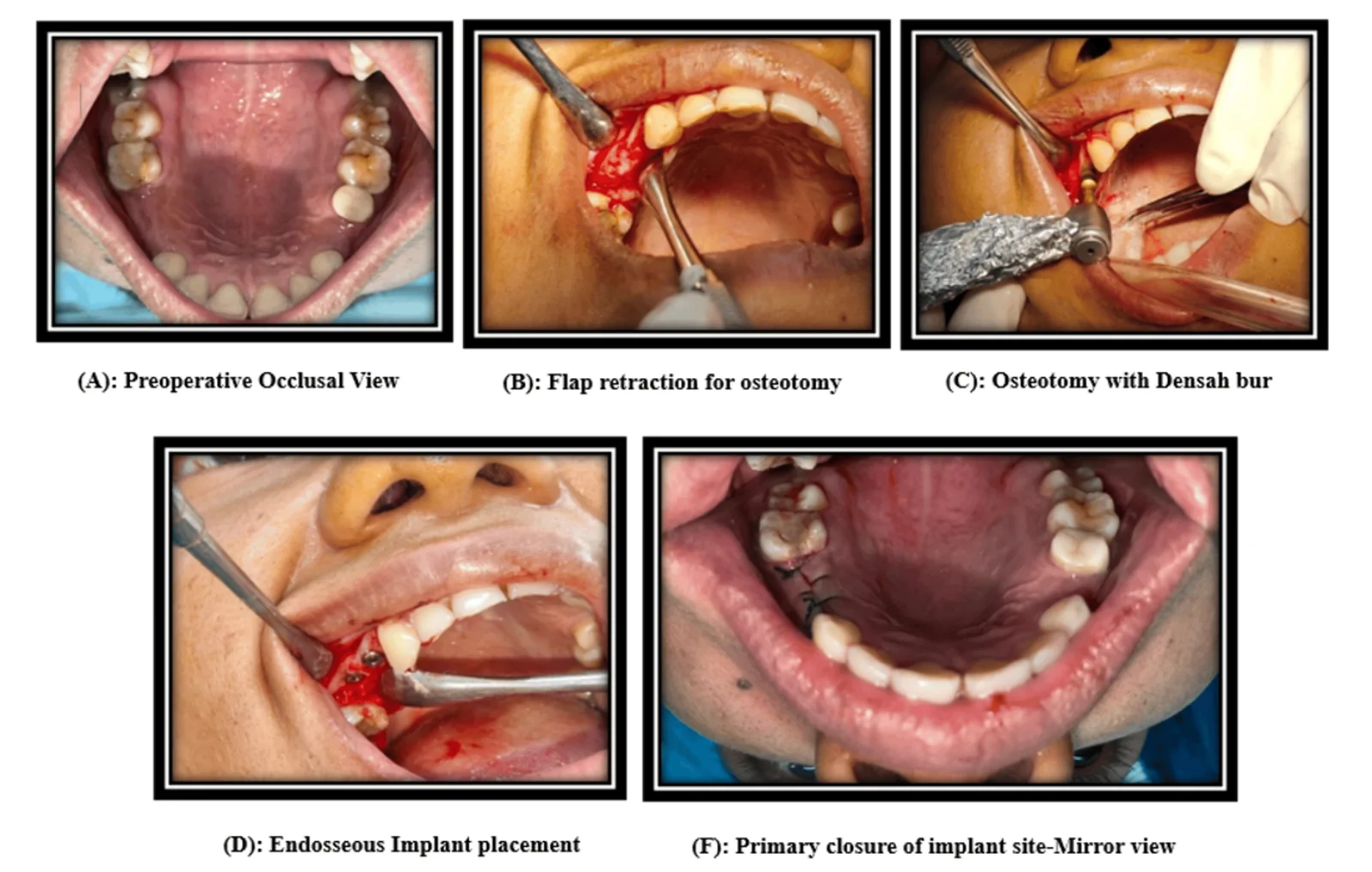
Clinical sequence showing implant osteotomy preparation and implant placement using the osseodensification protocol. (A) Preoperative occlusal view. (B) Flap retraction for osteotomy. (C) Osteotomy with Densah bur. (D) Endosseous implant placement. (E) Primary closure after implant placement.

The implant osteotomy was prepared according to the osseodensification protocol using Densah burs. Osteotomy preparation was initiated using a pilot drill, followed by sequential use of Densah burs (2.0 mm, 2.5 mm, 3.0 mm, and 3.5 mm) in counterclockwise densifying mode. A pilot drill was used initially to establish the implant site, angulation, and depth. Sequential Densah burs were then used in a counterclockwise densifying mode at 800-1,500 rpm under copious irrigation. After completion of the osteotomy, endosseous root-form dental implants were placed. Postoperative care was provided according to routine institutional protocol, and patients were recalled for postoperative evaluation and radiographic follow-up (Figure 2).

Prosthetic rehabilitation was performed after an uneventful healing period of four to five months.

Outcome assessment

The primary outcome variables were peri-implant bone density and alveolar ridge width. Peri-implant bone density was measured in Hounsfield units, while alveolar ridge width was measured in millimeters. Both parameters were evaluated at the following three levels: L1, representing the crestal level; L2, representing the mid-apical level; and L3, representing the apical level. Measurements were performed at the following three time intervals: preoperative, immediate postoperative, and four months postoperative. The mean of L1, L2, and L3 was calculated for both peri-implant bone density and alveolar ridge width.

Statistical analysis

Descriptive statistics were calculated as mean and standard deviation. Intergroup comparisons between D3 and D4 bone were performed using independent t-tests. Intragroup comparisons between different time intervals were performed using paired t-tests. Correlation analysis was performed to evaluate the relationship of age with peri-implant bone density and alveolar ridge width. Gender-based comparisons were performed to assess possible differences between male and female subjects. A p-value <0.05 was considered statistically significant.

## Results

Demographic distribution

The study included 20 subjects, of whom seven were males and 13 were females. The D3 group included three males and seven females, while the D4 group included four males and six females. The mean age of the total sample was 41.3 ± 13.0 years. The mean age was 43.6 ± 11.6 years in the D3 group and 39.0 ± 14.5 years in the D4 group (Table 1).

**Table 1:** Demographic distribution of the study sample according to sample size, gender distribution, mean age, and age range in the overall sample, D3 bone group, and D4 bone group. SD = standard deviation

Group	Sample size	Males	Females	Mean age ± SD	Age range
Overall sample	20	7	13	41.3 ± 13.0 years	22–68 years
D3 bone group	10	3	7	43.6 ± 11.6 years	22–62 years
D4 bone group	10	4	6	39.0 ± 14.5 years	22–68 years

Peri-implant bone density

The preoperative mean peri-implant bone density was 481.5 ± 99.5 HU in D3 bone and 332.9 ± 78.1 HU in D4 bone, with a statistically significant difference between the groups (p = 0.002). Immediately after implant placement, the mean peri-implant bone density increased to 578.8 ± 150.0 HU in D3 bone and 466.7 ± 80.0 HU in D4 bone. The immediate postoperative difference between D3 and D4 bone remained statistically significant (p = 0.049). At the four-month postoperative interval, the mean peri-implant bone density further increased to 689.4 ± 193.7 HU in D3 bone and 588.7 ± 105.6 HU in D4 bone. Although D3 bone continued to show higher mean density values, the intergroup difference at four months was not statistically significant (p = 0.166) (Table 2).

**Table 2:** Intergroup comparison of mean peri-implant bone density between D3 and D4 bone at preoperative, immediate postoperative, and four-month postoperative intervals. Test used: independent-samples t-test. SD = standard deviation; HU = Hounsfield units; S = statistically significant (p < 0.05); NS = non-significant (p > 0.05)

Time interval	D3 bone, mean ± SD (HU)	D4 bone, mean ± SD (HU)	Mean difference (HU)	P-value	Significance
Preoperative	481.5 ± 99.5	332.9 ± 78.1	148.7	0.002	S
Immediate postoperative	578.8 ± 150.0	466.7 ± 80.0	112.2	0.049	S
Four months postoperative	689.4 ± 193.7	588.7 ± 105.6	100.6	0.166	NS

**Figure 3:**
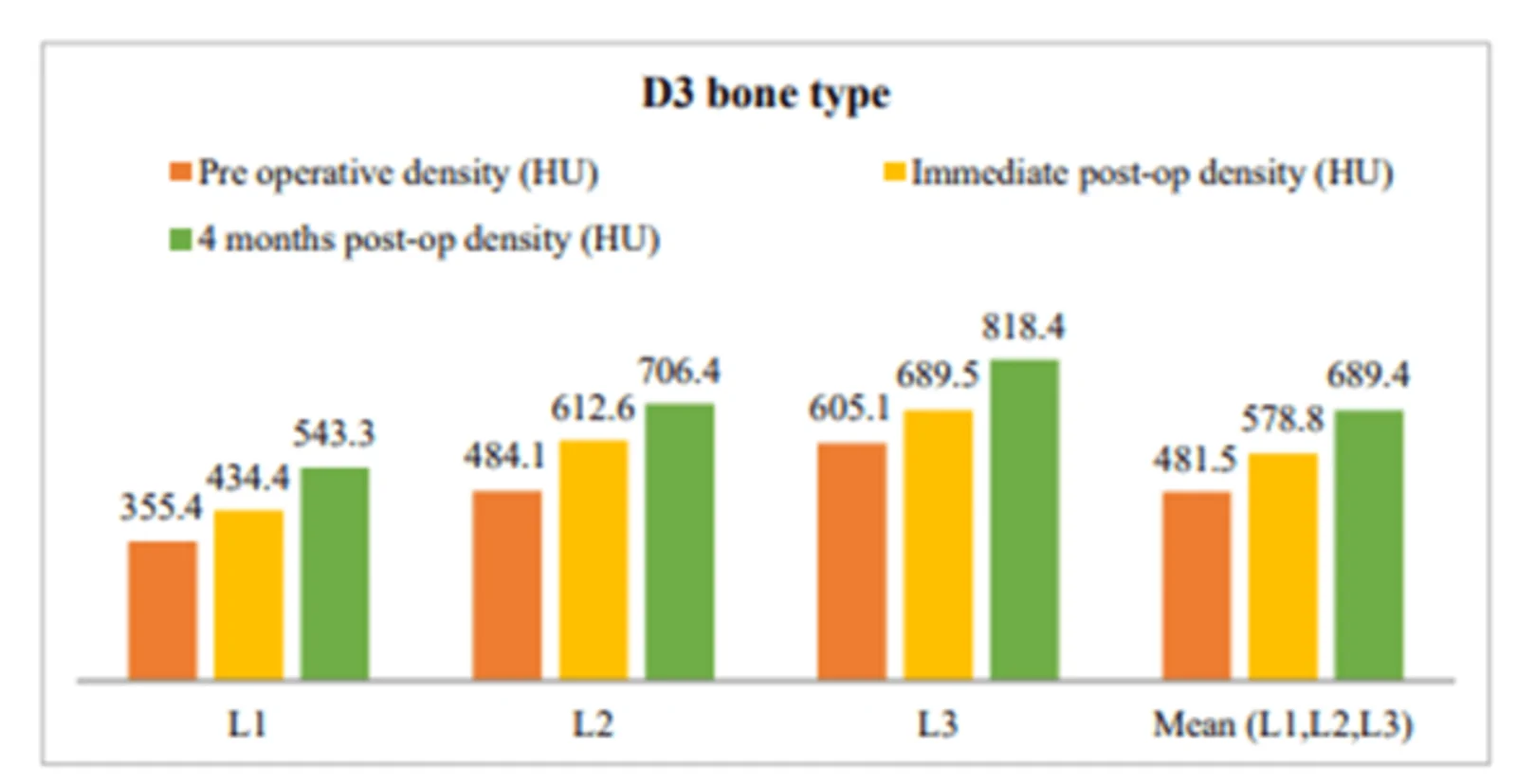
Bar chart showing peri-implant bone density changes in D3 bone at preoperative, immediate postoperative, and four-month postoperative intervals. HU = Hounsfield units; L1 = at the crestal level; L2 = 5 mm above L1; L3 = 5 mm above L2

Intragroup analysis showed a progressive increase in peri-implant bone density in both D3 and D4 bone groups. In D3 bone, the mean peri-implant bone density increased from 481.5 ± 99.5 HU preoperatively to 578.8 ± 150.0 HU immediately postoperatively and 689.4 ± 193.7 HU at four months (Figure 3).

**Figure 4:**
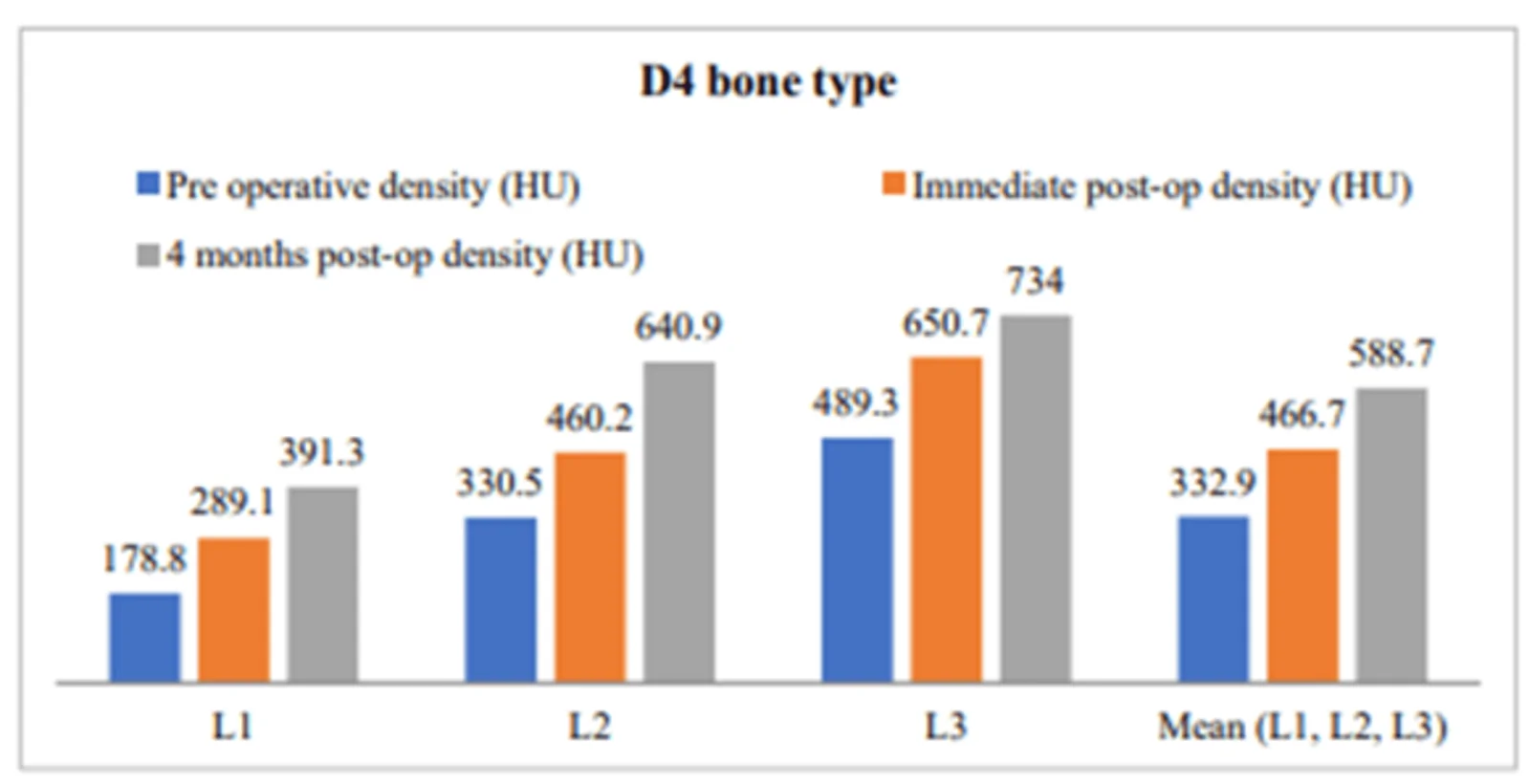
Bar chart showing peri-implant bone density changes in D4 bone at preoperative, immediate postoperative, and four-month postoperative intervals. HU = Hounsfield units; L1 = at the crestal level; L2 = 5 mm above L1; L3 = 5 mm above L2

In D4 bone, the corresponding increase was from 332.9 ± 78.1 HU preoperatively to 466.7 ± 80.0 HU immediately postoperatively and 588.7 ± 105.6 HU at four months (Figure 4).

These findings indicate that osseodensification produced a progressive improvement in peri-implant bone density in both bone types.

Alveolar ridge width

The mean alveolar ridge width increased in both groups after osseodensification. In D3 bone, the mean ridge width increased from 8.2 mm preoperatively to 8.9 mm immediately postoperatively and 9.8 mm at four months. In D4 bone, the mean ridge width increased from 8.0 mm preoperatively to 8.5 mm immediately postoperatively and 8.8 mm at four months. Although D3 bone showed a slightly greater increase in ridge width compared with D4 bone, the intergroup differences were not statistically significant (Table 3).

**Table 3:** Intergroup comparison of mean alveolar ridge width between D3 and D4 bone at preoperative, immediate postoperative, and four-month postoperative intervals. Test used: independent-samples t-test. SD = standard deviation; NS = statistically non-significant (p > 0.05)

Time interval	D3 bone, mean ± SD (mm)	D4 bone, mean ± SD (mm)	Mean difference (mm)	P-value	Significance
Preoperative	8.2 ± 2.4	8.0 ± 1.7	0.2	0.802	NS
Immediate postoperative	8.9 ± 2.1	8.5 ± 1.5	0.5	0.571	NS
Four months postoperative	9.8 ± 2.3	8.8 ± 1.4	0.9	0.291	NS

Level-wise assessment showed that ridge width gain was more pronounced at the mid-apical and apical levels than at the crestal level. In D3 bone, ridge width at L1 increased from 7.0 mm preoperatively to 7.9 mm at four months, while L2 increased from 8.3 mm to 10.1 mm and L3 increased from 9.3 mm to 11.3 mm. In D4 bone, ridge width at L1 increased from 7.0 mm to 7.2 mm, while L2 increased from 7.8 mm to 8.9 mm and L3 increased from 9.1 mm to 10.4 mm. These findings show that the dimensional gain was greater in the deeper trabecular regions than in the crestal region (Table 4).

**Table 4:** Level-wise alveolar ridge width changes in D3 and D4 bone at L1, L2, L3, and mean levels across preoperative, immediate postoperative, and four-month postoperative intervals. P-values represent intergroup comparison between D3 and D4 bone by independent-samples t-test. L1 = at the crestal level; L2 = 5 mm above L1; L3 = 5 mm above L2; NS = non-significant (p > 0.05)

Level	D3 preoperative width (mm)	D4 preoperative width (mm)	P-value	Inference	D3 immediate postoperative width (mm)	D4 immediate postoperative width (mm)	P-value	D3 four-month width (mm)	D4 four-month width (mm)	P-value
L1	7.0	7.0	0.992	NS	7.0	7.1	0.889	7.9	7.2	0.363
L2	8.3	7.8	0.684	NS	9.3	8.6	0.475	10.1	8.9	0.198
L3	9.3	9.1	0.845	NS	10.5	9.8	0.409	11.3	10.4	0.483
Mean	8.2	8.0	0.802	NS	8.9	8.5	0.571	9.8	8.8	0.291

## Discussion

The present study evaluated the effect of osseodensification on peri-implant bone density and alveolar ridge width in D3 and D4 bone of the posterior maxilla. The posterior maxilla is clinically challenging because it commonly presents with low-density trabecular bone, reduced ridge dimensions, and limited mechanical resistance during implant insertion [4]. As primary stability is strongly influenced by local bone quality and density, improving the peri-implant bone environment is particularly important in D3 and D4 bone [3].

**Figure 5:**
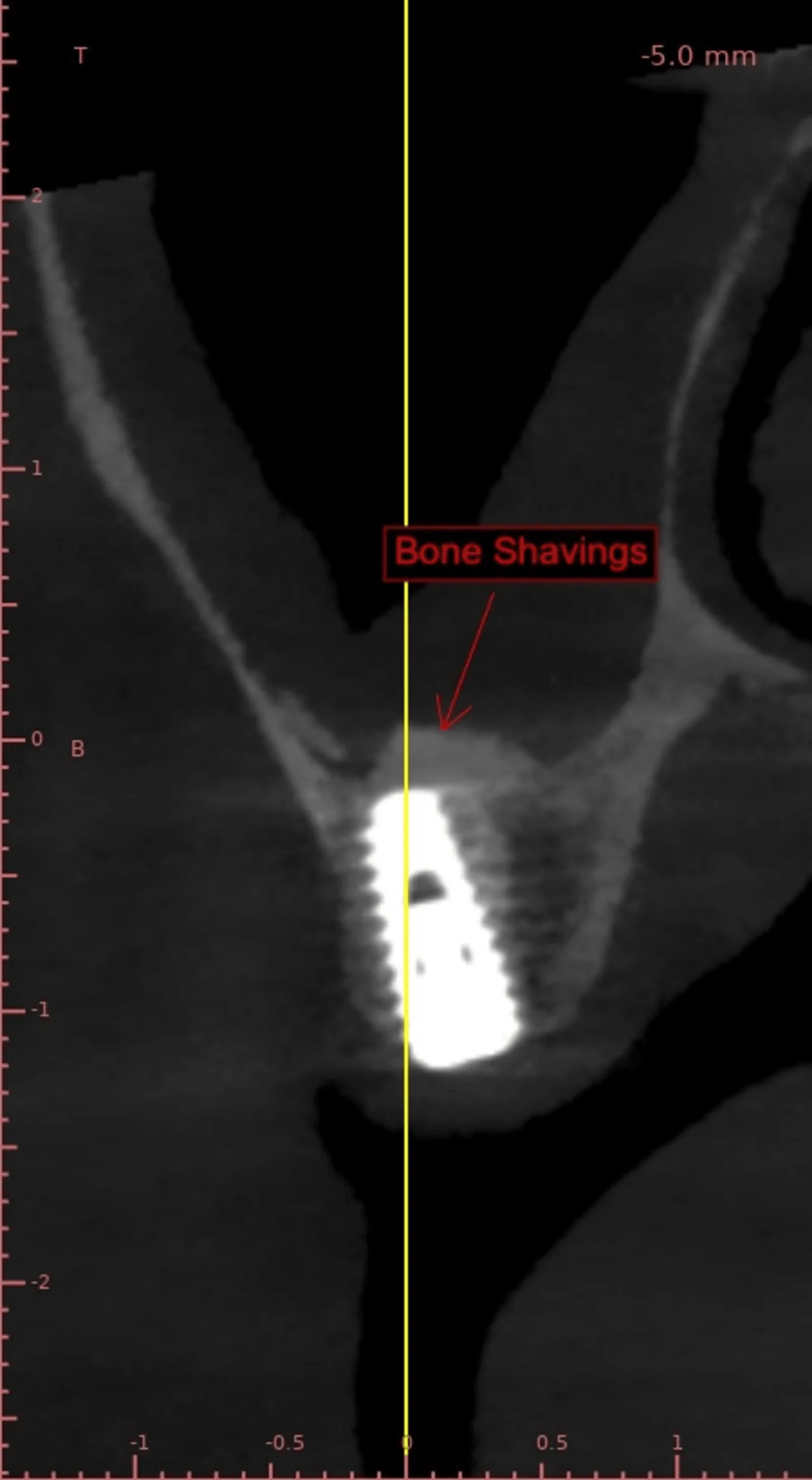
Representative immediate postoperative cone-beam computed tomography image demonstrating compacted autogenous bone chips (arrow) surrounding the implant following osseodensification.

The immediate postoperative CBCT demonstrated the presence of compacted autogenous bone chips surrounding the implant osteotomy. These preserved bone particles are characteristic of the osseodensification technique and may act as an autografting substrate, potentially contributing to increased peri-implant bone density and improved osseointegration observed during the healing period (Figure 5).

The results of the present study showed a progressive increase in peri-implant bone density in both D3 and D4 bone groups following osseodensification. This supports the principle that osseodensification preserves and compacts bone rather than removing it during osteotomy preparation [7]. In D3 bone, the mean peri-implant bone density increased from 481.5 ± 99.5 HU preoperatively to 689.4 ± 193.7 HU at four months. In D4 bone, the mean density increased from 332.9 ± 78.1 HU preoperatively to 588.7 ± 105.6 HU at four months. This increase indicates that osseodensification produced both an immediate densifying effect and continued improvement during the healing period.

Lahens et al. reported improved biomechanical fixation and increased bone-to-implant contact after osseodensification in low-density bone [8]. Bandela et al. reported improved primary stability with osseodensification compared with conventional osteotomy preparation in an ex vivo study [5]. Aloorker et al. also observed improvement in bone density and maintenance of crestal bone levels after osseodensification in a split-mouth clinical study [11]. The findings of the present study are consistent with these observations, as both D3 and D4 bone showed increased peri-implant bone density after osseodensification.

An important finding of this study was that D3 bone showed significantly higher bone density than D4 bone at baseline and immediately after implant placement, but the difference was no longer statistically significant at four months. This suggests that osseodensification may exert a particularly beneficial effect in poorer quality bone. Although D3 bone had higher absolute density values, D4 bone demonstrated a substantial relative improvement over time. Clinically, this may be relevant because D4 bone is generally more compromised and more difficult for achieving predictable implant stability.

The present study also showed measurable alveolar ridge expansion in both bone groups. In D3 bone, mean ridge width increased from 8.2 mm preoperatively to 9.8 mm at four months, while in D4 bone, it increased from 8.0 mm to 8.8 mm. Koutouzis et al. demonstrated that osseodensification can produce alveolar ridge expansion through plastic deformation and compaction autografting [9]. Salman and Bede reported that osseodensification enabled ridge expansion and implant placement in narrow alveolar ridges without dehiscence or fenestration [10]. The present study supports these findings and shows that osseodensification can produce clinically measurable dimensional changes in posterior maxillary bone.

The level-wise findings showed that ridge expansion was more pronounced at L2 and L3 than at L1. This may be explained by the difference in bone architecture at different levels of the ridge. The crestal region contains relatively more cortical bone and may resist deformation, whereas the mid-apical and apical regions contain more trabecular bone and may respond better to lateral compaction produced by densifying burs. This observation is clinically important because it indicates that osseodensification may not produce uniform expansion throughout the entire ridge, and clinicians should interpret crestal and deeper ridge changes separately.

Age and gender did not show a consistent statistically significant influence on peri-implant bone density or alveolar ridge width. This suggests that the observed improvements were mainly associated with the local effect of osseodensification and the baseline bone characteristics rather than demographic variables. However, this finding should be interpreted with caution because of the limited sample size.

The statistically significant increase in alveolar ridge width and peri-implant bone density observed in the present study supports the effectiveness of the osseodensification technique in D3 and D4 bone of the posterior maxilla. The preservation and compaction of autogenous bone during osteotomy preparation may contribute to increased peri-implant bone density and improved healing. These findings are supported by the statistically significant differences observed between the study time points and are consistent with the biological principles of osseodensification, which promotes bone preservation and compaction rather than bone excavation.

The present study has several strengths. It evaluated the effect of osseodensification in the clinically challenging D3 and D4 bone of the posterior maxilla, with serial CBCT assessments performed at preoperative, immediate postoperative, and four-month follow-up intervals. Furthermore, alveolar ridge width was assessed at three standardized levels (L1, L2, and L3), providing a comprehensive evaluation of ridge expansion and peri-implant bone density changes. To minimize methodological variability, all radiographic measurements were performed by a single examiner using a standardized protocol. In addition, the same implant system and dimensions (Dentis System, 4.0 × 10 mm) were used in all patients, thereby minimizing implant-related variability.

However, the present study also has certain limitations. The relatively small sample size and the absence of a conventional osteotomy control group limit the generalizability of the findings and preclude direct comparison with conventional drilling techniques. Furthermore, the increase in peri-implant bone density observed during the follow-up period may have been influenced not only by the osseodensification technique but also by the normal physiological bone healing process. In addition, peri-implant bone density was assessed using CBCT gray-scale values, which may not directly correspond to Hounsfield units obtained from medical CT, and the lack of calibration may introduce variability in density estimation. Therefore, the findings should be interpreted with this limitation in mind.

The study evaluated peri-implant bone density during the healing phase before functional loading; therefore, changes following prosthetic loading were not assessed. Furthermore, Implant Stability Quotient measurements were not included in the study protocol. These factors should be considered when interpreting the findings. Future randomized controlled clinical trials with larger sample sizes, longer follow-up periods, post-loading assessment, and additional clinical outcome measures are recommended.

## Conclusions

Within the limitations of this study, osseodensification significantly improved peri-implant bone density in both D3 and D4 bone of the posterior maxilla. The technique also produced measurable alveolar ridge expansion, with greater dimensional gain at the mid-apical and apical levels than at the crestal level. D3 bone showed higher baseline and postoperative density values than D4 bone, but the difference between the groups reduced by the four-month postoperative interval. Age and gender did not significantly influence the measured outcomes. Osseodensification appears to be a useful implant osteotomy technique for low-density posterior maxillary bone because it preserves bone, enhances peri-implant density, and promotes controlled ridge expansion. Larger controlled clinical studies with longer follow-up are required before broad clinical recommendations can be made.
